# Amaurotic Family History

**Published:** 1906-05-05

**Authors:** 


### AMAUROTIC FAMILY IDIOCY.

A strange disease, and full of suggestiveness as to complexity of the physical nature of the human being, is amaurotic family idiocy. That a child should be born healthy, and after a few months of life begin to show signs of degeneration of special character, is mysterious, but that this form of degeneration should be almost entirely limited to infants born of the Jewish race is still more mysterious. We recognise differences in moral character in men of different nationality, but we are less ready to believe that there is something within their physical blood, if we may so express it, that marks one race off from another. A recent paper of M. Sarvonat,[1] who has collected records of 74 cases of amaurotic family idiocy, and finds that all but three were in Jewish children, adds confirmation to the observed racial character of this disease. That the affection should run in families is, perhaps, less surprising, but nevertheless an interesting feature. M. Sarvonat refers to a family tree published by Falkenheim showing that cases were known to have occurred in three generations. The first case of this variety of idiocy was seen at the London Hospital by Mr. Warren Tay, and was described by him as "Symmetrical Changes in the Region of the Yellow Spot in an Infant." This description indicates the most characteristic feature of the disease; but it was not for affection of the eyesight that the child was taken to Mr. Warren Tay. As with most other recorded cases, it was the weakness of the child which attracted the attention of the mother. Infants affected with this disease in the first few months after birth are bright, and full of such life and vigour as babies then usually possess. At some time during the first year they begin, however, to be less easily interested, may fail to hold articles placed in their hands, or be unable to sit up or hold up the head. To this increasing weakness are added later spasmodic contractures of the muscles. Although muscular weakness may first attract attention, that the power of vision is also affected generally becomes apparent to the parents, and, when medical advice is sought, the ophthalmoscope reveals the appearances first described by Mr. Warren Tay—namely, a grey area in the neighbourhood of the fovea centralis, surrounded, it may be, by a few vessels, and in the midst of the greyish area a brownish red spot. The optic nerve also gradually atrophies. The loss of power, loss of eyesight, with the spasdomic contractures which follow, end in death, usually before the second year. M. Sarvonat seems to think that the appearance of this form of degeneration in the Jewish race is due to the fact that they are generally poorer and less developed physically than the white races amongst whom they live. We, in England, are certainly familiar with Jews that are neither endowed with average physique nor with too many of the necessities of life, yet, curiously enough, there are fewer wasting babies amongst their offspring than amongst those born of English mothers. To us it seems that there are no grounds for considering that inability of Jewish mothers to nourish their babies is a cause of amaurotic family idiocy.

[1] Revue Mensuelle des Maladies de l'enfance.

